# The ‘White-eyed’ Orbital Blowout Fracture: An Easily Overlooked Injury in Maxillofacial Trauma

**DOI:** 10.7759/cureus.4412

**Published:** 2019-04-09

**Authors:** Nicholas P Saggese, Ebrahim Mohammadi, Vito A Cardo

**Affiliations:** 1 Oral and Maxillofacial Surgery, Brookdale University Hospital and Medical Center, Brooklyn, USA; 2 Oral and Maxillofacial Surgery, Babol University of Medical Science, Babol, IRN

**Keywords:** white-eyed, orbital trauma, missed injury, misdiagnosis, maxillofacial trauma, muscle entrapment, lamina papyracea, oculocardiac reflex, orbital blowout fracture, diagnostic pitfall

## Abstract

The ‘white-eyed’ blowout fracture (WEBOF) is an injury that is often overlooked in head trauma patients, as it often has few overt clinical and radiographic features. Although benign in appearance, it can lead to significant patient morbidity. Here, we intend to increase the awareness of WEBOF and provide general principles for its diagnosis. WEBOF should be recognized early to ensure timely management and a successful outcome.

## Introduction and background

Orbital wall fractures can lead to serious patient morbidity, including the oculocardiac reflex (also known as Aschner phenomenon or trigeminocardiac reflex), diplopia, enophthalmos, hypoglobus, infraorbital nerve paresthesia, and ophthalmoplegia [1]. In the setting of orbital trauma, orbital wall fractures have a reported incidence of 4% to 70% [2]. Clinical diagnosis can be difficult, especially when there are no other facial fractures present to increase the chance of ecchymosis and edema. Therefore, it is essential to assess the eyes of all head trauma patients carefully upon presentation to the emergency department (ED).

Jordan et al. were the first to report that orbital fractures may lack external signs of injury, and coined the term white-eyed blowout fracture (WEBOF) in 1998 [3]. WEBOF is most commonly seen in the pediatric population and was originally defined as an orbital blowout fracture with the following characteristics: 1) discrete evidence of fracture on imaging studies; 2) restriction in ocular motility; and 3) minimal to no soft tissue trauma, hence the term “white-eye” [3]. Consequently, these orbital fractures often have a delayed diagnosis or even go unnoticed. Moreover, patients with WEBOF can present with bradycardia, headache, and malaise (secondary to the oculocardiac reflex) mimicking an intracranial injury, which can distract the clinician from the true etiology [4]. A comprehensive ocular examination and basic imaging modalities can be used to screen for the presence of WEBOF. Prompt diagnosis is crucial as surgery and medical management should proceed in a timely manner to avoid complications that can significantly affect the quality of life of the patient. This review is intended to increase the awareness of this injury and stress the importance of a thorough ocular examination in all patients with recent head trauma.

## Review

**Figure 1:**
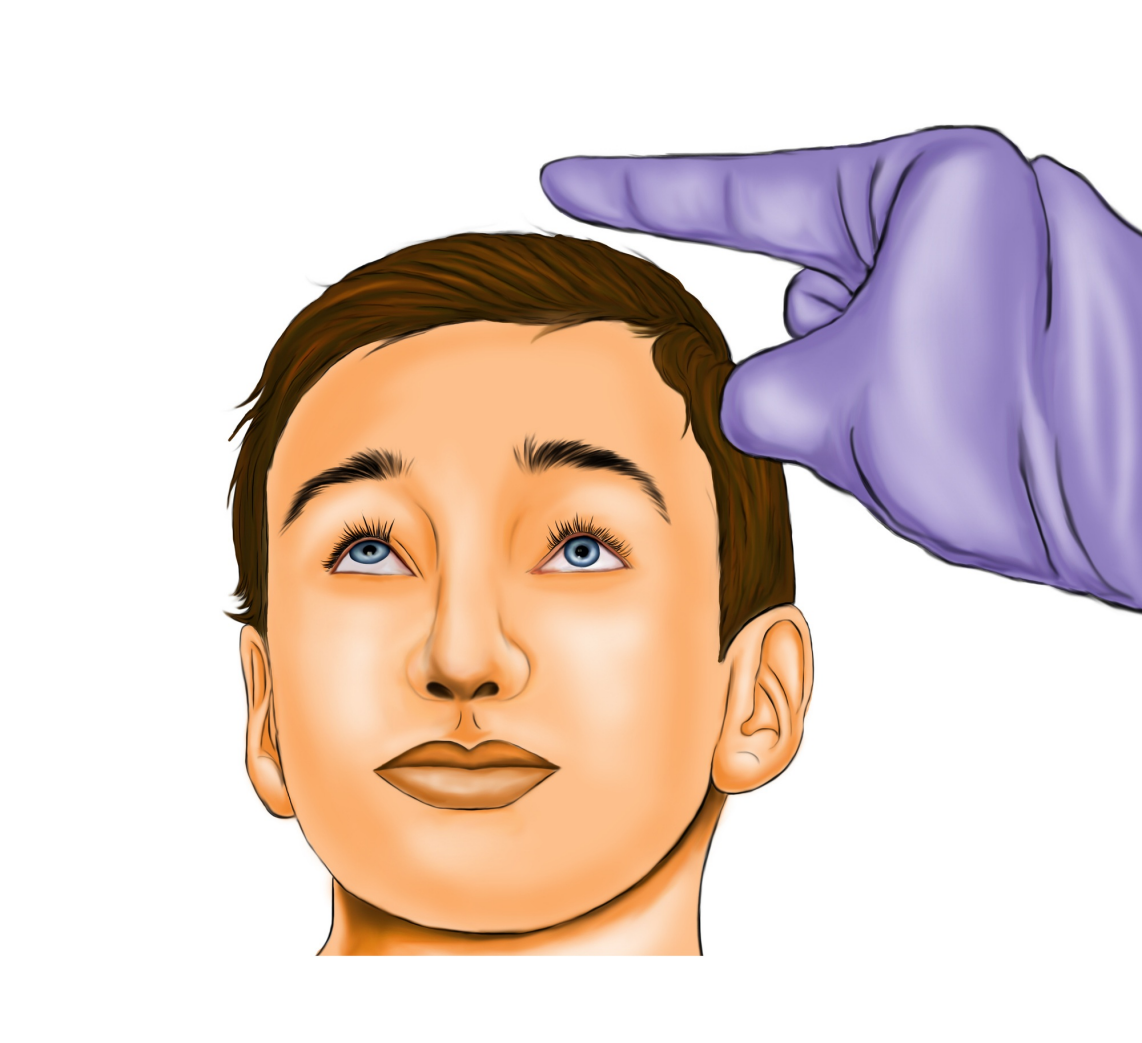
Left-sided WEBOF in a child. Note that the left eye exhibits restricted upward gaze (suggesting inferior rectus muscle entrapment) and there are no signs of external soft tissue trauma. WEBOF: White-eyed blowout fracture

A literature review revealed that WEBOFs are more prevalent in the pediatric population and are commonly overlooked in the ED. Moreover, there is a correlation between early surgical intervention and a favorable outcome [4-7]. Although WEBOF is more common in pediatric patients (Figure 1), it can also occur in adults. Ethunandan and Evans reported seven cases of WEBOFs, of which one was a 29-year-old [8]. Mehanna et al. reported a 21-year-old man with WEBOF that underwent surgery six days after the injury [9]. This patient continued to have extraocular muscle paresis, despite satisfactory postoperative computed tomography of the maxillofacial bones (CTMF). Therefore, WEBOFs can occur in the adult as well as pediatric population, and early surgical management may play a crucial role in both age groups.

Multiple authors reported missed WEBOFs, which led to poor outcomes. Hammond et al. reported a 14-year-old boy in whom a left WEBOF was mistaken for a head injury, delaying diagnosis [4]. Unfortunately, diplopia and limited ocular movement were not fully corrected in this patient even though surgery was performed 60 hours after the incident. If the WEBOF had not been misdiagnosed, surgery could have been performed earlier and morbidity in this young patient may have been prevented. According to Cobb et al., one-third of children are erroneously admitted for head injury observation when the true etiology is an orbital blowout fracture [10]. Jurdy and Malhotra reported another WEBOF case that was mistaken for a head injury [7]. The patient presented with diplopia along with oculocardiac reflex symptoms, such as bradycardia, nausea, and vomiting. The CTMF was initially misinterpreted as normal when in reality there was a left medial wall fracture. Referral to the surgical team was delayed by three days due to this CTMF misreading. Fortunately, oculocardiac symptoms resolved and the patient regained full ocular motility postoperatively. Mehanna et al. reported three cases of WEBOF, of which one was initially mistaken for a head injury and another was completely missed [9]. Regrettably, the patient with the missed orbital fracture exhibited persistent extraocular muscle paresis despite adequate surgical reconstruction and no muscle impingement evident on postoperative CTMF. This paresis may have been due to muscle necrosis secondary to the late diagnosis and management. Shamir et al. reported two cases of missed WEBOFs in young women who were victims of a suicide bomber terrorist attack [11]. Entrapment was not detected until both patients presented two weeks later with complaints of visual discomfort.

Considering that most of these missed injuries presented with autonomic symptoms, WEBOF should be included in the differential diagnosis for head trauma patients who present with bradycardia and/or a recent history of nausea or vomiting. As revealed by the literature review, these symptoms may be misleading, suggesting intracranial injury. Furthermore, these reports suggest that many physicians may be unaware of the WEBOF and of the appropriate examination to ascertain its presence. Diplopia, painful gaze, limited ocular motility, or autonomic symptoms should raise suspicion of WEBOF. If a low suspicion for WEBOF exists and radiation is a concern, then cone beam computed tomography (CBCT) can be considered for further investigation, as CBCT involves 42% less radiation than conventional computed tomography [12].

Although some orbital fractures have no indications for surgery, they still require medical management. Ben Simon et al. found orbital cellulitis to be rare, occurring in only 0.8% of their patients with orbital blowout fractures [13]. However, this condition can have severe complications, including cavernous sinus thrombosis, meningitis, blindness, brain abscess, septicemia, and death [14-16]. Therefore, WEBOF patients should avoid blowing their nose or sneezing through their nose, as this could force sinus bacteria into the surrounding tissues, which could ultimately cause infection or increased intraocular pressure [17]. The Valsalva maneuver should also be avoided, for the same reason. This is especially problematic in scuba divers who often use this technique to equalize their ear pressure [17]. Elevated intraocular pressure due to air forced into the orbit can lead to blindness. Moreover, blood pressure should be closely monitored because hypertension can lead to increased bleeding inside and around the ocular compartment. Likewise, patients on blood thinners should be observed for orbital hematoma [17]. The incidence of retrobulbar hematoma after facial trauma is less than 1%, but can cause blindness in up to 48% of cases [18]. Furthermore, the oculocardiac reflex has been shown to occur in fractures without entrapment [19]. Therefore, it is important to evaluate for bradycardia in all orbital fractures, regardless of the presence of entrapment. Bradycardia can be treated with a vagolytic agent, such as glycopyrrolate or atropine.

WEBOF may be difficult to identify on imaging. Tse et al. reported the case of a 14-year-old male who sustained a WEBOF from an elbow blow to the face [20]. Since the CTMF showed a small fracture and there were minimal signs of soft tissue trauma, the seriousness of the injury was overlooked and the patient was given a plastic surgery outpatient appointment one week later. Upon follow-up, he was urgently taken to the operating room for orbital exploration and reconstruction due to decreased visual acuity and signs of entrapment. Fortunately, diplopia and gaze restriction resolved postoperatively, but this case illustrates how the seriousness of this injury can be confused due to its benign appearance on imaging and a suboptimal physical examination. In one study, a CTMF detected soft tissue entrapment in only nine of 21 operative blowout fractures [21]. Heggie et al. suggested that entrapment is mainly a clinical diagnosis and that magnetic resonance imaging (MRI) should be considered in unclear cases, as it is more sensitive for soft tissue entrapment than CTMF [22]. Pediatric orbital fractures can be particularly difficult to interpret on CTMF; therefore, MRI can play a pivotal role as an adjunctive investigation [22, 23]. Nevertheless, the financial burden versus the benefit of an MRI should be weighed in this decision. Limited ocular movement in facial trauma patients who have no other physical examination findings can be almost pathognomonic for WEBOF [22]. This stresses the importance of a comprehensive ocular examination for these patients.

It is common for pediatric patients with blowout fractures to present with nausea and vomiting. Yew et al. reported a case of WEBOF in which the boy vomited twice after the injury [6]. Mehanna et al. reported three cases of WEBOF that all presented with symptoms of nausea [9]. Heggie et al. reported 22 cases of isolated blowout fractures, in which 10 presented with nausea and vomiting [22]. Two-thirds of these cases were WEBOFs [13]. Other studies have reported an incidence of autonomic symptoms of 21-28% in pediatric patients with orbital fractures [24-26]. Therefore, it is important to ask the patient or guardian about nausea or vomiting after the incident, which may provide a clue about a possible orbital fracture. Nausea and vomiting is likely due to the oculocardiac reflex, triggered by the traction of intraorbital tissues. The elasticity of the thin medial and orbital floors allows the fracture to snap back into place, trapping the rectal muscle inside the corresponding paranasal sinus [5, 7, 13].

Although surgical intervention guidelines are somewhat controversial, it is generally agreed in the literature that intervention for WEBOFs with entrapment should be performed within 48 hours to prevent Volkmann's contracture of the extraocular muscles, persistent oculocardiac reflex symptoms, and diplopia [3, 22, 27-32]. However, some authors had success with surgery at a later stage. For example, Yew et al. performed surgery, with successful outcomes, 72 hours after the injury, while Shamir et al. performed surgery two weeks after the injury, with success [6, 11]. Tse et al. reported five WEBOF cases in which only two had complete resolution of symptoms [20]. These two cases underwent orbital reconstruction at two weeks and at 16 hours, respectively. Two other cases, one that underwent surgery at day 19 and another that underwent surgery at day nine, had persistent diplopia and restricted gaze, perhaps due to the later intervention. The fifth case underwent no surgery and exhibited persistent diplopia on lateral gaze at one-year follow-up. Ethunandan and Evans reported two patients who were operated late, at 11 days and 20 days after the injury, both of whom had residual diplopia despite surgery [8]. The patient who was operated on at the 11th day underwent re-exploration and diplopia was subsequently resolved. Overall, the literature suggests that early surgical intervention is more successful, and that the chance for symptom resolution decreases with time [3, 33]. Therefore, if either entrapment or diplopia exists, we suggest that surgery should be performed within 48 hours. If the oculocardiac reflex is present, surgery should be performed immediately to prevent mortality. Taken together, immediate diagnosis is critical to afford the patient the best chance for recovery, and medical management is required for non-operative cases.

**Figure 2:**
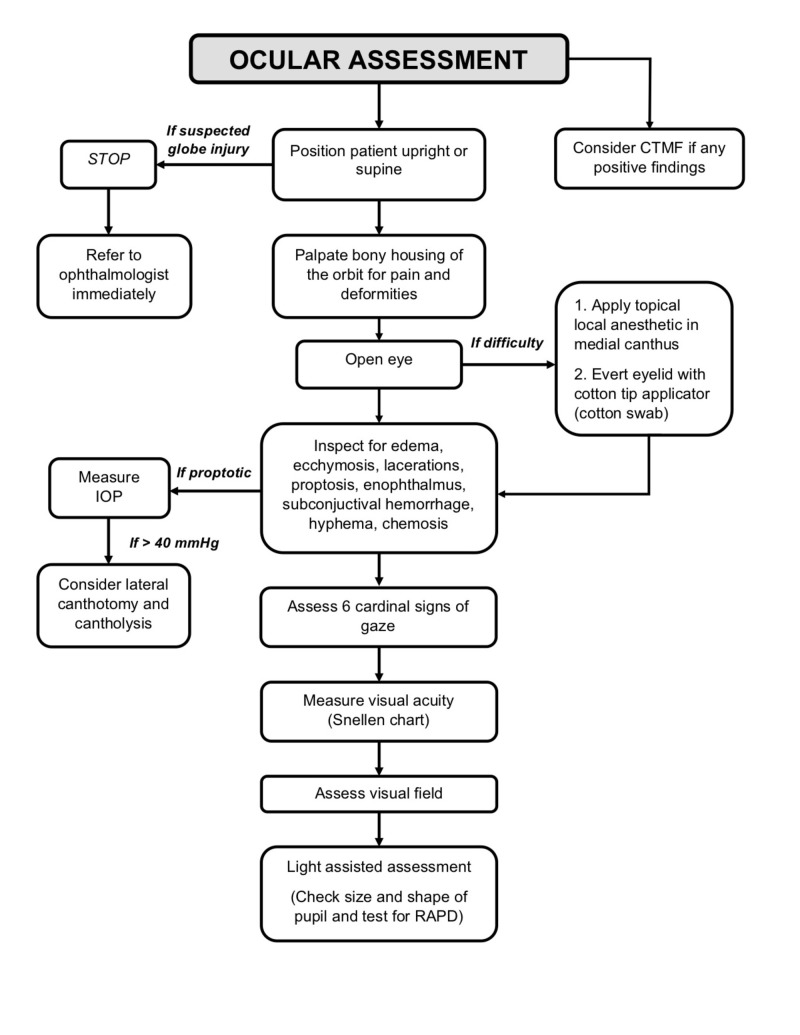
Flow diagram of the basic ophthalmologic examination required for head trauma patients. CTMF: Computed tomography of the maxillofacial bones; IOP: Intraocular pressure; RAPD: Relative afferent pupillary defect.

The WEBOF can be difficult to diagnose due to minimal external signs of soft tissue trauma and due to non-displaced fractures on CTMF. We recommend that all patients with a recent history of head trauma undergo a close examination of the orbits, including evaluation of periorbital tissues, infraorbital nerve function, pain on palpation, pulse rate on attempted gaze, ocular motility, visual acuity, visual fields, and pupillary responses to rule out injury to the orbit (Figure 2) [34]. Additionally, all patients should be asked if they have had recent nausea or vomiting. If mechanical entrapment is suspected, thin-slice CTMF should be performed along with a forced duction test performed under anesthesia. This test should be done when extraocular muscle entrapment is suspected. The limbus is gripped with toothed forceps and the globe is rotated in all directions. Limited movement in any direction would indicate entrapment of the respective rectus muscle. If the CTMF is negative for fractures and muscle incarceration, but WEBOF is still suspected due to limited ocular gaze, MRI should be considered for a more detailed overview of the soft tissues.

## Conclusions

WEBOF is a rare injury that can easily be overlooked due to subtle clinical and radiographic findings. Accordingly, all head trauma patients should be thoroughly evaluated for its presence. Surgery should be performed immediately in the presence of entrapment and autonomic symptoms. Medical intervention, such as sinus precautions, should be provided for patients with non-operative orbital fractures. Delayed diagnosis of WEBOF can lead to extraocular muscle necrosis, permanent diplopia, blindness, restricted gaze, and life-threatening oculocardiac reflex complications. Therefore, all physicians who deal with head trauma should have a high index of suspicion for this benign-appearing, yet serious injury.

## References

[1] O'Connell JE, Hartnett C, Hickey-Dwyer M, Kearns GJ (2015). Reconstruction of orbital floor blow-out fractures with autogenous iliac crest bone: a retrospective study including maxillofacial and ophthalmology perspectives. J Craniomaxillofac Surg.

[2] Morris CD, Tiwana PS (2012). Diagnosis and treatment of midface fractures. Oral and Maxillofacial Trauma.

[3] Jordan DR, Allen LH, White J, Harvey J, Pashby R, Esmaeli B (1998). Intervention within days for some orbital floor fractures: the white-eyed blowout. Ophthalmic Plast Reconstr Surg.

[4] Hammond D, Grew N, Khan Z (2013). The white-eyed blowout fracture in the child: beware of distractions. J Surg Case Rep.

[5] Lane K, Penne RB, Bilyk JR (2007). Evaluation and management of pediatric orbital fractures in a primary care setting. Orbit.

[6] Yew CC, Shaari R, Rahman SA, Alam MK (2015). White-eyed blowout fracture: diagnostic pitfalls and review of literature. Injury.

[7] Jurdy L, Malhotra R (2011). White-eyed medial wall blowout fracture mimicking head injury due to persistent oculocardiac reflex. J Craniofac Surg.

[8] Ethunandan M, Evans BT (2011). Linear trapdoor or “white-eye” blowout fracture of the orbit: not restricted to children. Br J Oral Maxillofac Surg.

[9] Mehanna P, Mehanna D, Cronin A (2009). White-eyed blowout fracture: another look. Emerg Med Australas.

[10] Cobb ARM, Jeelani NO, Ayliffe PR (2013). Orbital fractures in children. Br J Oral Maxillofac Surg.

[11] Shamir D, Ardekian L, Peled M (2008). Blowout fracture of the orbit as a result of blast injury: case report of a unique entity. J Oral Maxillofac Surg.

[12] Brisco J, Fuller K, Lee N, Andrew D (2014). Cone beam computed tomography of imaging orbital trauma—Image quality and radiation dose compared with conventional multislice computed tomography. Br J Oral Maxillofac Surg.

[13] Ben Simon GJ, Bush S, Selva D, McNab AA (2005). Orbital cellulitis: a rare complication after orbital blowout fracture. Ophthalmology.

[14] Ferguson P, McNab A (1999). Current treatment and outcome in orbital cellulitis. Clin Exp Ophthalmol.

[15] Chaudhry A, Shamsi A, Elzaridi E (2007). Outcome of treated orbital cellulitis in a tertiary eye care center in the Middle East. Ophthalmology.

[16] Shuttleworth GN, David DB, Potts MJ, Bell CN, Guest PG (1999). Orbital trauma: do not blow your nose. BMJ.

[17] Ellis E III (2012). Orbital trauma. Oral Maxillofac Surg Clin North Am.

[18] Fattahi T, Brewer K, Retana A, Ogledzki M (2014). Incidence of retrobulbar hemorrhage in the emergency department. J Oral Maxillofac Surg.

[19] Woernley TC, Wright TL, Lam DN, Jundt JS (2017). Oculocardiac reflex in an orbital fracture without entrapment. J Oral Maxillofac Surg.

[20] Tse R, Allen L, Matic D (2007). The white-eyed medial blowout fracture. Plast Reconstr Surg.

[21] Parbhu K, Galler K, Li C, Mawn L (2008). Underestimation of soft tissue entrapment by computed tomography in orbital floor fractures in the pediatric population. Ophthalmology.

[22] Heggie AA, Vujcich NJ, Shand JM, Bordbar P (2015). Isolated orbital floor fractures in the paediatric patient: case series and review of management. Int J Oral Maxillofac Surg.

[23] Schmutz B, Rahmel B, McNamara Z, Coulthard A, Schuetz M, Lynham A (2014). Magnetic resonance imaging: an accurate, radiation-free, alternative to computed tomography for the primary imaging and three-dimensional reconstruction of the bony orbit. J Oral Maxillofac Surg.

[24] Bansagi Z, Meyer D (2000). Internal orbital fractures in the pediatric age group: characterization and management. Ophthalmology.

[25] Egbert JE, May K, Kersten RC, Kulwin DW (2000). Pediatric orbital floor fracture: direct extraocular muscle involvement. Ophthalmology.

[26] Cohen S, Garrett C (2003). Pediatric orbital floor fractures: nausea/vomiting as signs of entrapment. Otolaryngol Head Neck Surg.

[27] Wei LA, Durairaj VD (2011). Pediatric orbital floor fractures. J Am Assoc Pediatr Ophthalmol Strabismus.

[28] Maloney K (2014). Non-displaced pediatric orbital fracture with displacement of the inferior rectus muscle into the maxillary sinus: a case report and review of the literature. Int J Oral Maxillofac Surg.

[29] Gerber B, Kiwanuka P, Dhariwal D (2013). Orbital fractures in children: a review of outcomes. Br J Oral Maxillofac Surg.

[30] Gerbino G, Roccia F, Bianchi FA, Zavattero E (2010). Surgical management of orbital trapdoor fracture in a pediatric population. J Oral Maxillofac Surg.

[31] Criden MR, Ellis FJ (2007). Linear nondisplaced orbital fractures with muscle entrapment. J Am Assoc Pediatr Ophthalmol Strabismus.

[32] Phan LT, Piluek WJ, McCulley TJ (2012). Orbital trapdoor fractures. Saudi J Ophthalmol.

[33] Grant JH, Patrinely JR, Weiss AH, Kierney PC, Gruss JS (2002). Trapdoor fracture of the orbit in a pediatric population. Plast Reconstr Surg.

[34] Mutie D, Mwangi N (2015). Assessing an eye injury patient. Community Eye Health.

